# Malignancy Rate of Indeterminate Findings on FDG-PET/CT in Cutaneous Melanoma Patients

**DOI:** 10.3390/diagnostics11050883

**Published:** 2021-05-15

**Authors:** Ken Kudura, Florentia Dimitriou, Daniela Mihic-Probst, Urs J. Muehlematter, Tim Kutzker, Lucas Basler, Robert Förster, Reinhard Dummer, Joanna Mangana, Lars Husmann, Irene A. Burger, Michael Christoph Kreissl

**Affiliations:** 1 Department of Nuclear Medicine, University Hospital Zurich, 8006 Zurich, Switzerland; urs.muehlematter@usz.ch (U.J.M.); lars.husmann@usz.ch (L.H.); irene.burger@usz.ch (I.A.B.); 2 Faculty of Medicine, University of Zurich, 8091 Zurich, Switzerland; florentia.dimitriou@usz.ch (F.D.); daniela.mihic@usz.ch (D.M.-P.); lucas.basler@psi.ch (L.B.); robert.foerster@ksw.ch (R.F.); reinhard.dummer@usz.ch (R.D.); johanna.mangana@usz.ch (J.M.); 3 Department of Dermatology, University Hospital Zurich, 8091 Zurich, Switzerland; 4 Institute of Pathology and Molecular Pathology, University Hospital Zurich, 8091 Zurich, Switzerland; 5 Faculty of Applied Statistics, Humboldt University Berlin, 10117 Berlin, Germany; tim.kutzker@hu-berlin.de; 6 Center for Proton Therapy, Paul Scherrer Institute, 5232 Villigen, Switzerland; 7 Institute of Radiation Oncology, Cantonal Hospital Winterthur, 8400 Winterthur, Switzerland; 8 Division of Nuclear Medicine, Department of Radiology and Nuclear Medicine, University Hospital Magdeburg, 39120 Magdeburg, Germany; michael.kreissl@med.ovgu.de

**Keywords:** positron emission tomography, computed tomography, melanoma, immunotherapy

## Abstract

Background: The use of ^18^*F*-2-Fluor-2-desoxy-D-glucose Positron Emission Tomography/Computed Tomography FDG-PET/CT in clinical routine for staging, treatment response monitoring and post treatment surveillance in metastatic melanoma patients has noticeably increased due to significant improvement of the overall survival rate in melanoma patients. However, determining the dignity of the findings with increased metabolic activity on FDG-PET/CT can be sometimes challenging and may need further investigation. Purpose: We aimed to investigate the malignancy rate of indeterminate findings on FDG-PET/CT in metastatic cutaneous melanoma patients. Methods: This single-center retrospective study included cutaneous melanoma patients who underwent FDG-PET/CT in clinical routine between 2015 and 2017 with findings reported as indeterminate and therefore requiring further evaluation. The dignity of the included findings was determined by subsequent imaging and, if required, additional histopathology. The impact of the outcome on the clinical management was also reported. Results: A total of 842 FDG-PET/CT reports of 244 metastatic cutaneous melanoma patients were reviewed. Sixty indeterminate findings were included. Almost half of all indeterminate findings were lymph nodes, lung nodules and cerebral lesions. In total, 43.3% of all included findings proved to be malignant. 81% of all malignant lesions were metastases of cutaneous melanoma, while 19% of all malignant lesions could be attributed to other primary malignancies, such as lung, breast, thyroid and colorectal cancers. Malignant findings influenced clinical management in 60% of the cases. Conclusion: Indeterminate findings on FDG-PET/CT in metastatic cutaneous melanoma patients should be further investigated. Almost one out of every two indeterminate findings on FDG-PET/CT is malignant. The majority of the findings are melanoma manifestations, however, in a significant percentage, other primary tumors are found. Upon verification, patient management is changed in most cases.

## 1. Introduction

Cutaneous melanoma causes 90% of skin cancer mortality [1] and is the leading cause of skin cancer-related mortality [2]. Over the past decades, the incidence of cutaneous melanoma has increased in many Western countries, affecting both younger and older populations [2,3], and becoming the most rapidly increasing cancer in Caucasian populations [4]. The number of cases of cutaneous melanoma is predicted to continue to increase for decades, particularly in Caucasians with excessive sun exposure [1]. However, significant improvements in the diagnosis and treatment of cutaneous melanoma have changed the context of this disease [5]. Immune checkpoint inhibitors (ICI), including anti-PD1 and anti-CTLA4, as well as targeted therapy with BRAF/MEK inhibitors for BRAF mutant disease have significantly prolonged the survival of patients with metastatic melanoma and are therefore deemed standard of care [6]. Due to the prolonged, sustainable responses, imaging-based surveillance of advanced stage III/IV disease is essential for early assessment of treatment efficacy, as well as locoregional and distant metastasis [3].

^18^*F*-2-Fluor-2-desoxy-D-glucose Positron Emission Tomography/Computed Tomography (FDG-PET/CT) has shown an additional value over Computed Tomography (CT) for staging in melanoma patients [7]. According to the 2019 European consensus-based interdisciplinary guideline update for cutaneous melanoma, whole-body FDG-PET/CT examinations in combination with brain MRI are recommended from stage IIC to stage IV (8th version of the American Joint Committee on Cancer AJCC classification) [1]. FDG-PET/CT is considered an effective tool for staging and treatment response assessment in metastatic melanoma patients [8]. However, a low specificity for initial locoregional lymph node metastases in early stages was observed [9].

Due to the mechanism of action of immune checkpoint inhibitors, the use of conventional morphological criteria such as Response Evaluation Criteria in Solid Tumors (RECIST) can be challenging. FDG-PET/CT can be used to assess the treatment response using morphologic and metabolic response criteria adapted to immunotherapy [10]. In order to correctly assess response to treatment, atypical response patterns such as pseudoprogression or hyperprogression [11,12,13,14] and immune-related adverse events [15,16,17] have to be taken into account.

After surgical or systemic treatment, a structured follow-up by FDG-PET/CT is required for early detection of relapses and secondary primary melanomas [1,2], although a consensus is still lacking regarding the timing of FDG-PET/CT [7]. A stage-based follow-up scheme by FDG-PET/CT from stage IIC to stage IV (8th version of AJCC classification) is recommended by the European guidelines 2019 [1].

Taking into consideration the significant improvement of the overall survival in melanoma patients [10], and the inclusion of FDG-PET/CT into the European guidelines 2019 for cutaneous melanoma, FDG-PET/CT has been increasingly used for staging, treatment response monitoring and post treatment surveillance in advanced melanoma [18]. Furthermore, given the known atypical response patterns under immunotherapy [11,12,13,14], a differentiation between increased metabolic activity due to tumor progression and increased metabolic activity due to, e.g., the effects of immune checkpoint inhibitors on FDG-PET/CT, can be challenging. It therefore appears relevant to quantify the malignancy risk of indeterminate findings reported on the FDG-PET/CT in metastatic cutaneous melanoma patients in clinical routine, and so further evaluation is required. Therefore, we aimed to investigate the malignancy rate of indeterminate findings on FDG-PET/CT at staging, treatment response assessment and post treatment surveillance after the required follow-up.

## 2. Methods

### 2.1. Patient Population

This study cohort included patients with histopathologically proven cutaneous melanoma treated at the Department of Dermatology of the University Hospital Zurich in Switzerland who underwent FDG-PET/CT as part of clinical routine for staging and/or treatment response assessment and/or post treatment surveillance.

This single-center retrospective study was approved by the local ethics committee (PB_2017-00181) and conducted in compliance with Good Clinical Practice (GCP) rules and the Declaration of Helsinki.

### 2.2. FDG-PET/CT Acquisition

All FDG-PET/CT scans were performed as part of clinical routine between 1 January 2015 and 31 December 2017 at the Department of Nuclear Medicine of the University Hospital Zurich, Switzerland, according to the department’s standard protocol.

Patients were asked to fast at least for 4 h prior the intravenous ^18^*F*-FDG-administration. A blood glucose level below 160 mg/dl at the time of ^18^*F*-FDG injection was mandatory. Image acquisition began 60 min after the administration of a body mass index (BMI)-adapted ^18^*F*-FDG dose. All examinations were performed on General Electric GE Discovery MI (DMI) and Discovery 690 PET/CT scanners (General Electrics GE Healthcare, Boston, MA, USA).

In accordance with our protocol, FDG-PET/CT scans were performed from the vertex of the skull to the thighs. Whole body FDG-PET/CT scans were performed only if the primary melanoma was located in the lower extremities. A CT scan without contrast medium was performed first for attenuation correction (matrix size 512 × 512; field of view 50 cm; slice thickness 3.75 mm) and immediately followed by the PET acquisition in six or seven bed positions (patient size adapted), with 2.5 min per bed position (matrix size 256 × 256; field of view 70 cm) using iterative reconstructions (Ordered Subset Expectation Maximization (OSEM); Block Sequential Regularized Expectation Maximation (BSREM)).

### 2.3. FDG-PET/CT Image Analysis

The analysis of fused FDG-PET/CT data displayed in transversal, sagittal and coronal planes was not part of our investigation but was previously carried out in clinical routine by experienced physicians board certified in radiology and nuclear medicine using GE Advantage Workstations 4.4-7.

### 2.4. Lesion Selection

We retrospectively reviewed the reports of all FDG-PET/CT scans performed in clinical routine between 1 January 2015 and 31 December 2017. A lesion was considered as indeterminate on the FDG-PET/CT whenever it could not be unequivocally characterized as benign nor malignant by the reporting physician and additional imaging was recommended for further evaluation.

Qualitative criteria were used in the reviewed FDG-PET/CT reports by the reporting nuclear physicians for the assessment of indeterminate findings.

Among our qualitative criterias for the assessment of indeterminate findings either the evidence of focal, not physiological, FDG-uptake without a clearly identifiable morphological correlate (in brain, bone, colon, kidney, liver, prostate, small bowel) or focal, not physiological, FDG-uptake with a subtle morphological correlation (in breast, cutis/subcutis, head/neck, mediastinum, thyroid gland). The following criteria were used for lymph nodes: a focal FDG-uptake without any clearly suspicious morphological criterion on native Computed Tomography scan (i.e., enlarged in short axis diameter, round in shape and no internal fat attenuation) or one morphological suspicious criterion without focal suspicious FDG-uptake. Small solid lung nodules with subtle FDG-uptake were included. The following morphological criteria were used with regards to solid lung nodules on native Computed Tomography scan: sharp margins (no lobulation, no spiculation), no calcification and smaller than 6 mm in size.

We subsequently recorded if the imaging requested by the reporting nuclear physician (i.e., ultrasound guided biopsy, mammography, computed tomography, magnetic resonance imaging, colonoscopy and bronchoscopy) was performed. Therefore, all reports from the interdisciplinary tumor board for melanoma, as well as any imaging performed subsequently between the date of the corresponding FDG-PET/CT scan and 30 November 2020, were reviewed. The recommended biopsy (guided by ultrasound, bronchoscopy, colonoscopy or after mammography) was performed 26.7 ± 19.1 days (average ± standard deviation) after the corresponding indeterminate finding was reported on the FDG-PET/CT scan, while a radiological follow-up (Computed Tomography or Magnetic Resonance Imaging) was carried out 75.9 ± 49.9 days (average ± standard deviation) after the FDG-PET/CT scan.

Each included indeterminate lesion was then classified as benign or malignant based on the results of subsequent imaging and, when available, additional histopathology.

Given the short time window of radiological follow-up, we decided to put the results of additional imaging and histopathology into the clinical context and investigate in close cooperation with the Department of Dermatology of the University Hospital Zurich if the result of the required evaluation had a long-term impact on clinical management (i.e., until 30 November 2020). This evaluation was based on clinical reports and interdisciplinary tumor board decisions.

### 2.5. Statistical Analysis

All statistical computations were performed using the Statistical Package for the Social Sciences software (SPSS Statistics Version 25.0.0.1; International Business Machines Corporation IBM, Armonk, NY, USA). Descriptive statistical analyses were carried out. Chi-square tests were used for the calculation of association between our parameters. Statistical significance was accepted at *p* < 0.05.

## 3. Results

### 3.1. Patient Population

The reports of 842 FDG-PET/CT scans performed in clinical routine between 1 January 2015 and 31 December 2017 for staging and/or treatment response assessment and/or post treatment surveillance at the Department of Nuclear Medicine of the University Hospital Zurich were retrospectively reviewed.

Among these 842 FDG-PET/CT reports of 244 metastatic melanoma patients, 90 indeterminate findings were observed, although in some cases no documentation on the recommended imaging could be noted retrospectively either because the required imaging after FDG-PET/CT was declined by the patient (*n* = 4) or, most probably, performed externally (*n* = 26).

**Figure 1:**
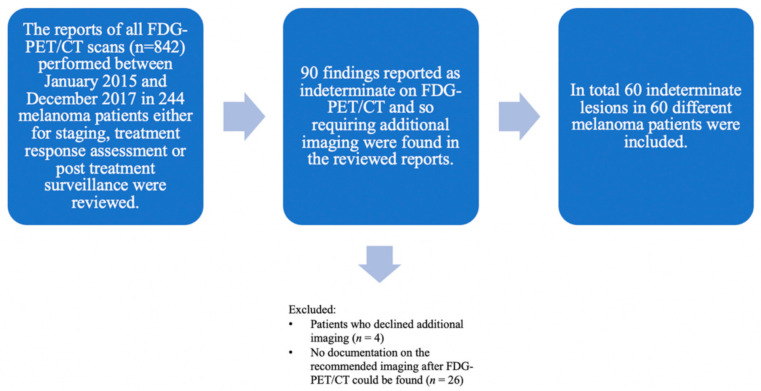
Flowchart showing lesion selection with all included and excluded lesions. The reports of all FDG-PET/CT scans (*n* = 842) performed between January 2015 and December 2017 in melanoma patients (*n* = 244) in clinical routine either for staging, treatment response assessment or post treatment surveillance were considered. Initially, 90 indeterminate findings on the FDG-PET/CT requiring additional imaging for further evaluation were retrospectively found in the reviewed reports. Lesions were excluded either when patients declined additional imaging (*n* = 4) or no documentation on the recommended imaging after FDG-PET/CT could be found (*n* = 26). In total, 60 indeterminate lesions were included. All included patients (*n* = 60) with histopathologically proven melanoma who were treated at the Department of Dermatology of the University Hospital Zurich—each patient with one indeterminate finding on the FDG-PET/CT.

Consequently, 60 indeterminate findings in 60 different melanoma patients (61.7% men, *n* = 37; 38.3% women, *n* = 23; mean age 67.4 years) were included. Each patient had only one finding on FDG-PET/CT requiring further imaging-based evaluation (Figure 1).

### 3.2. Indeterminate Findings on FDG-PET/CT Scan

**Figure 2:**
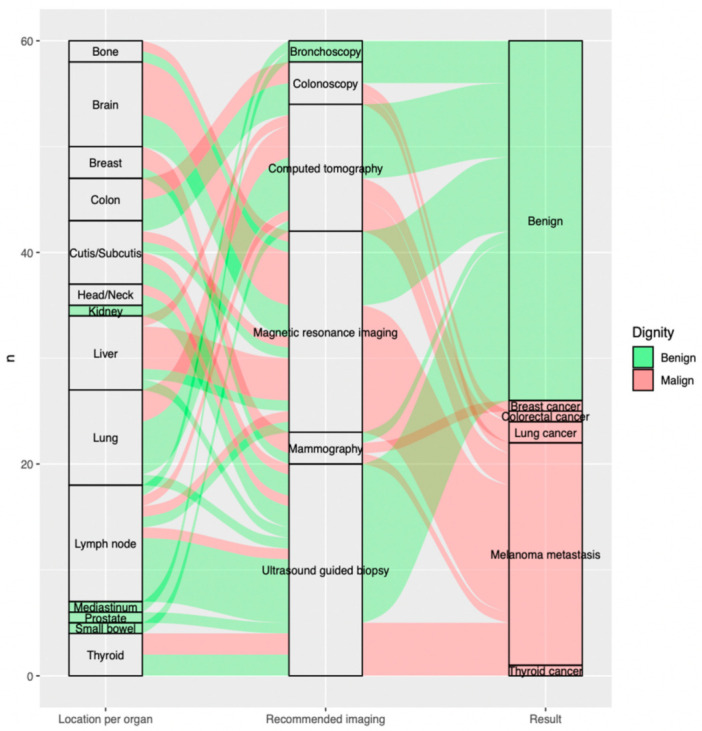
Sankey plot showing, per included indeterminate finding on the FDG-PET/CT (*n* = 60), on the far left, (**a**) location per organ; in the middle, (**b**) recommended imaging for further evaluation; and the far right, (**c**) outcome of additional imaging. Benign findings are displayed in green, malign findings in red arrows. (**a**) Location per organ (on the left from the bottom to the top): thyroid (*n* = 4; 5.0%, small bowel (*n* = 1; 1.7%), prostate (*n* = 1; 1.7%), mediastinum (*n* = 1; 1.7%), lymph node (*n* = 11; 18.3%), lung (*n* = 9; 15.0%), liver (*n* = 7; 11.7%), kidney (*n* = 1; 1.7%), head/neck (*n* = 2; 3.3%), cutis/subcutis (*n* = 6; 10.0%), colon (*n* = 4; 6.7%), breast (*n* = 3; 5.0%), brain (*n* = 8; 13.3%), bone (*n* = 2; 3.3%). (**b**) Recommended imaging (in the middle from the bottom to the top): ultrasound guided biopsy (*n* = 20; 33.3%), mammography (*n* = 3; 5.0%), magnetic resonance imaging (*n* = 19; 31.7%), computed tomography (*n* = 12; 20.0%), colonoscopy (*n* = 4; 6.7%), bronchoscopy (*n* = 2; 3.3%). (**c**) Result (on the right from the bottom to the top): thyroid cancer (*n* = 1; 1.7%), melanoma metastasis (*n* = 21; 35.0%), lung cancer (*n* = 2; 3.3%), colorectal cancer (*n* = 1; 1.7%), breast cancer (*n* = 1; 1.7%), benign (*n* = 34; 56.7%).

The most frequent indeterminate findings on the FDG-PET/CT were lymph nodes 18.3% (*n* = 11), lung nodules 15.0% (*n* = 9) and brain lesions 13.3% (*n* = 8) constituting altogether 46.6% (*n* = 28) of all included findings. The fourth and fifth most recurrent findings requiring further evaluation were liver lesions 11.7% (*n* = 7) and cutaneous/subcutaneous lesions 10.0% (*n* = 6) (Figure 2).

### 3.3. Additional Imaging Required after FDG-PET/CT Scan

The most commonly recommended additional imaging after FDG-PET/CT was ultrasound-guided biopsy 33.3% (*n* = 20), followed by magnetic resonance imaging 31.7% (*n* = 19) and computed tomography 20.0% (*n* = 12), which altogether account for 85% of all requested further examinations (Figure 2).

### 3.4. Standard Assessment of Dignity

The dignity of each included lesion was assessed based on radiological follow up (i.e., CT or MRI, *n* = 31, 51.6%) and, whenever available, histopathology (i.e., biopsy guided by ultrasound, colonoscopy, bronchoscopy or based on mammography, *n* = 29, 48.4%) (Table 1).

### 3.5. Outcome of Required Additional Imaging after FDG-PET/CT Scan

When assessing the malignancy rate of various organs, only 27.3% of the included indeterminate lymph nodes were found to be malignant, while 33.3% of the indeterminate lung nodules, 62.5% of the indeterminate cerebral lesions and 71.4% of the indeterminate liver lesions were classified as malignant. All indeterminate findings in the small bowel, kidney, mediastinum and prostate proved to be benign; however, 50% of the findings in the bone, colon and head/neck were malignant. Finally, 66.7% of the indeterminate findings located in the breast and 33.3% of the cutaneous or subcutaneous indeterminate findings were proven to be malignant. In total, 43.3% (*n* = 26) of all included findings were found to be malignant and 56.7% (*n* = 34) benign (Table 2).

Of all malignant lesions, 81% (*n* = 21) were metastatic melanoma lesions, while 19% were additional malignancies: 8% (*n* = 2) were found to be lung cancer, 4% (*n* = 1) breast cancer, 4% (*n* = 1) thyroid cancer and 4% (*n* = 1) colorectal cancer, respectively (Figure 2).

**Figure 3:**
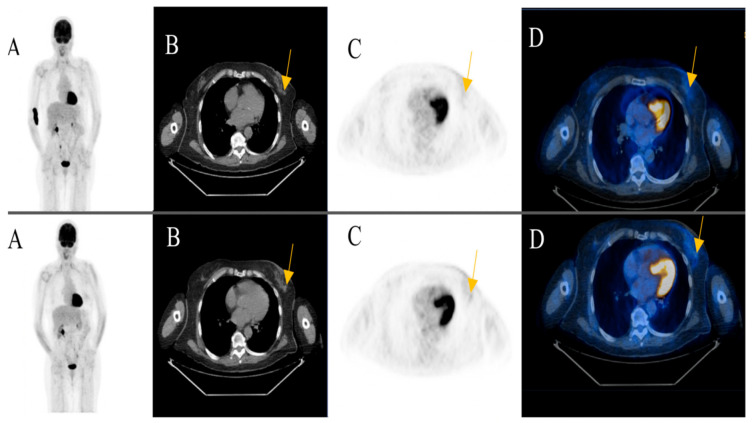
An 81-year-old female patient initially with metastatic cutaneous melanoma stage IV (pT2a N3 M1b). (**A**) Maximum intensity projection (MIP); (**B**) Computed Tomography (CT) axial of chest; (**C**) Positron Emission Tomography( PET) axial of chest; (**D**) Fused images PET/CT axial of chest. First row: FDG-PET/CT performed for surveillance after treatment without evidence of metabolically active recurrence, nodal or distant metastasis. Contamination at injection site cubital on the right upper extremity. Second row: Six months later, a morphologic and metabolic progredient subcutaneous nodule in the left breast was seen (yellow arrow). An ultrasound-guided biopsy was subsequently recommended for further evaluation. The biopsy revealed an invasive ductal breast cancer.

Among the patients with additional primary cancers, an indeterminate subcutaneous nodule was seen in the left breast of an 81-year-old female patient in post treatment surveillance (initially with metastatic cutaneous melanoma stage IV). The reporting physicians recommended an ultrasound-guided biopsy for further evaluation. The biopsy revealed an invasive ductal breast cancer (Figure 3) (Case 1).

**Figure 4:**
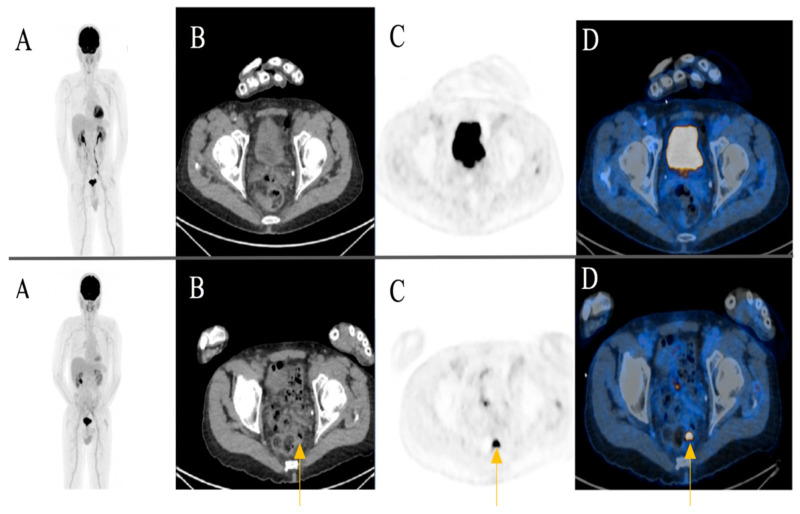
A 72-year-old male patient initially with metastatic cutaneous melanoma stage IIIC (T0 N2b M0). (**A**) Maximum intensity projection (MIP); (**B**) Computed Tomography (CT) axial of pelvis; (**C**) Positron Emission Tomography (PET) axial of pelvis; (**D**) Fused images PET/CT axial of pelvis. First row: FDG-PET/CT performed for extended staging after left axillary lymphadenectomy without evidence of metabolically active melanoma, nodal or distant metastasis. Second row: Follow-up for treatment response assessment under anti-PD1 monotherapy. Three months after treatment initiation, a new metabolically active wall thickening of the rectosigmoidal colon was seen (yellow arrow). A rectoscopy was subsequently recommended for further evaluation. The biopsy revealed a well-differentiated adenocarcinoma of the colon.

A focal FDG-uptake with wall thickening in the rectosigmoidal colon was also observed in a 72-year-old male patient with metastatic cutaneous melanoma stage IIIC during treatment response assessment. A rectoscopy was subsequently performed based on the recommendation of the reporting physicians. The biopsy revealed a well-differentiated adenocarcinoma of the colon (Figure 4) (Case 2).

### 3.6. Impact of Required Additional Imaging on Clinical Management

Overall, the outcome of additional imaging required after FDG-PET/CT influenced clinical management in 25% of all cases (*n* = 15, *p* = 0.00 < 0.05), leading either to initiation of a new treatment 18.3% (*n* = 11), abort of ongoing therapy 5.0% (*n* = 3) or a change in treatment 1.7% (*n* = 1).

**Figure 5:**
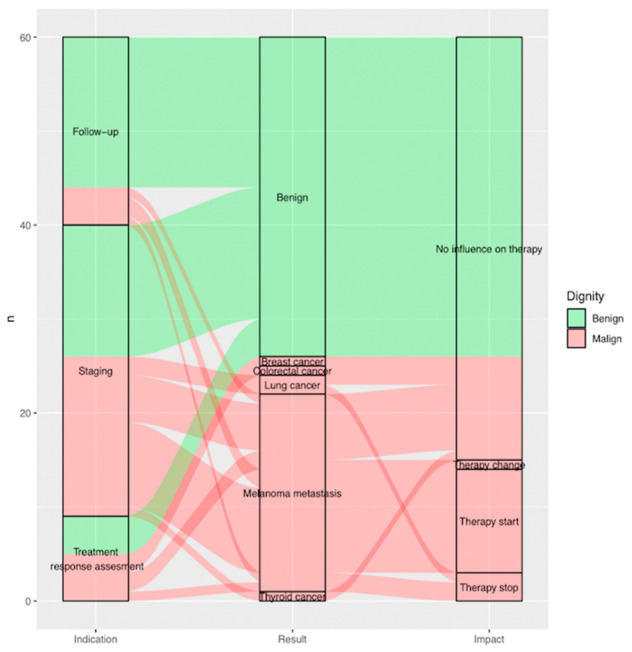
Sankey plot showing, per included indeterminate finding (*n* = 60), on the far left, (**a**) indication of FDG-PET/CT scan it was reported on as indeterminate; in the middle, (**b**) result of additional imaging; and on the far right, (**c**) impact of additional imaging on clinical management. Benign findings are displayed in green, malignant findings in red arrows. (**a**) Indication (on the left from the bottom to the top): treatment response assessment (*n* = 9; 15.0%), staging (*n* = 31; 51.7%) and follow-up (*n* = 20; 33.3%). (**b**) Result (in the middle from the bottom to the top): thyroid cancer (*n* = 1; 1.7%), melanoma metastasis (*n* = 21; 35.0%), lung cancer (*n* = 2; 3.3%), colorectal cancer (*n* = 1; 1.7%), breast cancer (*n* = 1; 1.7%), benign (*n* = 34; 56.7%). (**c**) Impact *(*on the right from the bottom to the top): therapy stop (*n* = 3; 5.0%), therapy start (*n* = 11; 18.3%), therapy change (*n* = 1; 1.7%) and no influence on therapy (*n* = 45; 75%).

Almost 60% (*n* = 15) of all findings found to be malignant after complementary evaluation (*n* = 26) showed an impact on clinical management (Figure 5) (Figure 3).

### 3.7. Impact of FDG-PET/CT Indication on Results of Recommended Additional Imaging

Of all FDG-PET/CT scans, 51.7% were performed for staging (*n* = 31), 33.3% (*n* = 20) for post treatment surveillance and 15.0% (*n* = 9) for treatment response assessment.

Of all indeterminate findings on FDG-PET/CT performed for staging, treatment response assessment and post treatment surveillance, 51.6%, 55.6% and only 20% were found to be malignant, respectively (*p* = 0.04 < 0.05). Of all malignant lesions, 64.0% (*n*=16) were found during staging. Eighty percent of findings (*n* = 16) reported as indeterminate on FDG-PET/CT during post treatment surveillance were found to be benign (Figure 3).

## 4. Discussion

This monocentric retrospective study was intended to investigate the malignancy rate of indeterminate findings on FDG-PET/CT along the pathway of metastatic melanoma patients at the levels of staging, treatment response assessment and post treatment surveillance.

Our results suggest that lymph nodes, lung nodules and brain lesions were the most frequent indeterminate findings on FDG-PET/CT. Ultrasound-guided biopsy, computed tomography and magnetic resonance imaging together accounted for 85% of all requested additional imaging after FDG-PET/CT.

A very significant association between indication of FDG-PET/CT and outcome of recommended imaging was observed. Almost half of the indeterminate findings on FDG-PET/CT during staging and treatment response assessment in melanoma patients were found to be malignant. The vast majority of malignant findings were melanoma metastasis, followed by additional primary tumors such as lung, breast, colon and thyroid cancer. Interestingly, most of the indeterminate findings on FDG-PET/CT during post treatment surveillance were found to be false positive, with lymph nodes and lung nodules being by far the most common false positive findings on FDG-PET/CT.

Our investigation also demonstrated a significant impact of the additional imaging required after FDG-PET/CT on clinical management in melanoma patients. Approximately 60% of all malignant findings lead either to treatment change, treatment stop or initiation of new therapy.

Alongside the benefits of imaging-based surveillance, potential false positive findings with additional healthcare management and costs in melanoma patients undergoing regular follow-ups have also been recently discussed [19]. Our first significant observation was that lymph nodes and lung nodules were the most frequent indeterminate findings on FDG-PET/CT that required additional imaging, but at the same time, were mostly false positives, particularly in post treatment surveillance. In 2019, Nijhuis et al., aimed to quantify false positive and incidental findings from annual surveillance imaging based on baseline CT or PET/CT and two annual surveillance scans. They observed 124 findings reported as suspicious of melanoma recurrence, and non-melanoma-related findings requiring further action in at least half of all 154 included stage III melanoma patients undergoing annual surveillance imaging. Interestingly, 88% (109 of 124) of these findings were found to be benign based on histopathology, subsequent imaging or clinical follow-up [19], which is in keeping with our results. A very significant association between the indication of FDG-PET/CT and the outcome of recommended imaging was observed in our population. Only 20% of all indeterminate findings reported on the FDG-PET/CT scans performed for post treatment surveillance were malignant in our cohort.

Our second important observation was that approximately 60% of all malignant findings (i.e., 25% of all included indeterminate findings on the FDG-PET/CT scans) led either to a change in treatment, stopping of treatment or the initiation of a new therapy. Pfannenberg et al., reported in 2019 that FDG-PET/CT had an impact on clinical management in 37.1% of cases, most frequently from nontreatment strategy to active treatment after FDG-PET/CT, with the highest rate of treatment changes found after FDG-PET/CT scans in melanoma patients (up to 46.0%) [20]. Forschner et al., also reported, based on prospective data published in 2017, that FDG-PET/CT prevented futile surgery by half (*n* = 55; 51.4%) of advanced (mainly stage III/IV) melanoma patients initially planned for radical metastasectomy [21]. The vast majority of malignant findings in our cohort were melanoma metastases, followed by additional primary tumors such as lung, breast, colon and thyroid cancer, particularly during staging and treatment response assessment. Dimitriou et al., described in 2019 hematologic or solid additional primary tumors (such as breast, thyroid cancer or leukemia) in melanoma patients as an important survival issue, which have emerged since the significant increase of life expectancy due to recent progress in diagnosis and treatment of melanoma [22].

Thirdly, we observed that ultimately only a few lesions were reported as indeterminate in all FDG-PET/CT reports reviewed (proportionally, in one report out of ten), suggesting additional imaging was often not required after FDG-PET/CT. Pfannenberg et al., reported in their prospective study published in 2019 that the greatest impact of FDG-PET/CT on clinical management of cancer patients was in reducing needs for further imaging and avoiding invasive tests by comparing intended management before and after FDG-PET/CT. Based on their German prospective registry data, additional imaging was initially required in 66.1% before FDG-PET/CT, and only 6.1% of cases after FDG-PET/CT. In addition, initially planned invasive tests could be avoided in 72.7% of cases after FDG-PET/CT. Alongside melanoma, Pfannenberg et al., considered several other malignancies such as lung cancer, lymphoma, prostate cancer and neuroendocrine tumors [20], while we focused solely on melanoma patients.

Of note, no contrast agent was used during the FDG-PET/CT acquisition according to our standard protocol for cutaneous melanoma in order to save radiation doses. As previously mentioned, lymph nodes, lung nodules, brain and liver were the most frequent locations for indeterminate findings on the FDG-PET/CT scan. Among the indeterminate liver lesions without evidence of malignancy, either hemangiomas or no morphological correlates were found. Meningiomas, gliotic changes or no morphological correlate were found among the indeterminate cerebral lesions without evidence of malignancy. The included lung nodules were round in shape with no spiculation or lobulation and ≤ 6mm in size. The considered lymph nodes were not enlarged and without central fat attenuation. After further evaluation, the pulmonal and lymphonodal indeterminate findings without evidence of malignancy were due to inflammatory changes. In light of these results, the injection of a contrast agent would presumably have, in few cases, provided additional information making a further workup, possibly including full-dose CT (e.g., for indeterminate findings in the liver such as hemangiomas) unnecessary. The potential additional morphological information provided by the administration of contrast media with regards to indeterminate findings in the small bowel, kidney and prostate, where all findings were found with no morphological correlate in our cohort, should be further investigated.

One possible limitation of our study could be the reproducibility of our results. Our investigations were carried out in a single university center with an interdisciplinary team and easy access to all imaging and interventional modalities for the evaluation of indeterminate findings. On the other hand, monocentricity offers advantages of similar image and report quality thanks to state-of-the-art equipment and experienced specialists, who weekly participate in a dedicated interdisciplinary tumor board for melanoma. Other limitations are the retrospective approach and size of our cohort, which could be overcome by further prospective investigations with larger groups in the future.

## 5. Conclusions

Indeterminate findings on FDG-PET (including low-dose CT) in metastatic cutaneous melanoma patients need to be further investigated. Almost one out of every two indeterminate findings, either on PET, low-dose CT or both, is malignant. The majority of the findings are melanoma manifestations; however, in a significant percentage, other primary tumors are found. Upon verification by follow-up imaging or biopsy, patient management is changed in most cases.

## Figures and Tables

**Table 1:** The dignity of all included lesions requiring further evaluation after the FDG-PET/CT scan (*n* = 60) was assessed based on radiological follow up (i.e., Computed Tomography (CT) or Magnetic Resonance Imaging (MRI)) and/or histopathology (i.e., biopsy guided by ultrasound, bronchoscopy, colonoscopy or based on mammography).

Organ	Radiological Follow Up (CT or MRI)	Biopsy Guide by Ultrasound	Biopsy Guide by ^a^ Bronchoscopy, ^b^ Colonoscopy or Based on ^c^ Mammography	Total
Lymph node	3	7	1a	11
Liver	6	1	0	7
Thyroid	0	4	0	4
Breast	1	1	1c	3
Cutis/Subcutis	3	3	0	6
Brain	8	0	0	8
Head/Neck	0	2	0	2
Lung	8	0	1a	9
Colon	0	0	4b	4
Small bowel	1	0	0	1
Bone	2	0	0	2
Kidney	0	1	0	1
Mediastinum	0	0	1a	1
Prostate	0	1	0	1
Total	31	20	9	60

The table shows, on a per organ basis, how often a radiological follow up and/or biopsy was recommended for all included lesions (*n* = 60). (c): The indication for ultrasound guided biopsy was not primarily based on FDG-PET/CT, but only secondarily after an abnormal recommended mammography in contrast to the other biopsies listed (*n* = 26), which were recommended based on FDG-PET/CT findings.

**Table 2:** All included lesions reported as indeterminate on FDG-PET/CT (*n* = 60) were classified as benign or malignant based on the outcome of the required further evaluation (i.e., radiological follow up and/or histopathology).

Organ	Number of Benign Finding(s) per Organ	Number of Malignant Finding(s) per Organ	Number of Indeterminate Finding(s) per Organ
Lymph node	8	3	11
Liver	2	5	7
Thyroid	2	2	4
Breast	1	2	3
Cutis/Subcutis	4	2	6
Brain	3	5	8
Head/Neck	1	1	2
Lung	6	3	9
Colon	2	2	4
Small bowel	1	0	1
Bone	1	1	2
Kidney	1	0	1
Mediastinum	1	0	1
Prostate	1	0	1
Total	34	26	60

The table shows the dignity of all included indeterminate findings per organ (first column from right), i.e., how often per considered organ (first column from left) an indeterminate finding on FDG-PET/CT was proved to be benign (second column from left) or malignant (third column from left) after further evaluation.

## Data Availability

All reviewed imaging modalities were performed during clinical routine. Patient data are stored in the local archiving system at the University Hospital Zurich, Switzerland.

## Abbreviations

ICI	Immune Checkpoint Inhibitors
anti-PD1	anti-Programmed Death-1
anti-CTLA4	anti-Cytotoxic T-Lymphocyte-Associated Protein 4
FDG-PET/CT	^18^*F*-2-Fluor-2-desoxy-D-glucose Positron Emission Tomography/Computed Tomography
CT	Computed Tomography
AJCC	American Joint Committee on Cancer
RECIST	Response Evaluation Criteria in Solid Tumors
GCP	Good Clinical Practice
mg	Milligram
dl	Deciliter
BMI	Body Mass Index
GE	General Electric
PET	Positron Emission Tomography
OSEM	Ordered Subset Expectation Maximization
BSREM	Block Sequential Regularized Expectation Maximation
SPSS	Statistical Package for the Social Sciences
MRI	Magnetic Resonance Imaging
DWI	Diffusion Weighted Imaging
MIP	Maximum Intensity Projection
